# Evaluating the Clinical Utility of MINDY in Monitoring Change and Risk on an Acute Psychiatric Ward

**DOI:** 10.1192/bjo.2026.11628

**Published:** 2026-06-30

**Authors:** Chania Lambrinudi, Alba Garcia-Melendez, Kishen Guruparan, Harith Ali, Kazuya Iwata

**Affiliations:** 1 East London NHS Foundation Trust, London, United Kingdom; 2 Queen Mary University London, London, United Kingdom

## Abstract

**Aims:**

Monitoring mental state and risk is central to psychiatric inpatient care. The narrative nature of the Mental State Examination (MSE), although essential for clinical decision-making, limits rapid detection of trends and communication of change across teams. Existingrating scales are often diagnosis-specific, time-consuming, or clinician-restricted, reducing feasibility for routine ward use.

MINDY is a brief, structured tool designed for repeated inpatient assessment to capture mental-state disturbance and risk in a reproducible format.

This study aimed to evaluate whether repeated MINDY assessments meaningfully reflect clinical change in mental state and risk during psychiatric admissions.

We hypothesised that MINDY scores and risk classifications would show clinically coherent variation over time and align with changes documented in nursing notes.

**Methods:**

This ward-based service-evaluation and retrospective case-note review was conducted on an adult psychiatric inpatient ward. Three resident doctors completed daily MINDY assessments for inpatients under a single consultant team over a randomly selected two-week period.

MINDY outputs were compared with concurrent nursing documentation. Data were anonymised and handled in accordance with NHS information-governance and General Data Protection Regulation requirements. Analyses were undertaken by an independent medical student with no prior clinical involvement.

The case-record analysis focused on MINDY’s capacity to track clinical change over time. Documentation completeness, inter-rater reliability and predictive validity were not assessed.

**Results:**

On days with both MINDY and nursing documentation available, suicide or self-harm risk was recorded on six occasions, all classified by MINDY as Risk-Group C (100% sensitivity and negative predictive value). Specificity was 87.5%, with three Risk-Group C classifications on days without documented risk. Agreement between Risk-Group C and nursing-documented risk was substantial (κ=0.74). Documented risk was significantly more frequent on Risk-Group C days than others (66.7% vs 0%), with a large effect size (h=1.91; p=0.00014).

Graphical longitudinal plots showed clinically interpretable trajectories. Patients discharged during the intervention recorded sustained low risk and scores, concordant with nursing notes, while those remaining on the ward showed fluctuating scores and shifting risk zones reflecting variable clinical courses.

**Conclusion:**

MINDY’s Risk-Group classification identified nursing-documented suicide and self-harm risk with perfect sensitivity and substantial agreement. Longitudinal visualisation demonstrated clinically meaningful trajectories associated with discharge and ongoing admission.

The findings support MINDY as a practical, structured tool for tracking inpatient change and guiding discharge decisions, though further refinement of risk stratification is needed.

